# Effect of hypothermia in patients undergoing simultaneous carotid endarterectomy and coronary artery bypass graft surgery

**DOI:** 10.5830/CVJA-2014-056

**Published:** 2015

**Authors:** Yucel Ozen, Eray Aksoy, Sabit Sarikaya, Ebuzer Aydin, Ozge Altas, Murat Bulent Rabus, Kaan Kirali

**Affiliations:** Kartal Kosuyolu Heart and Research Hospital, Istanbul, Turkey; Kartal Kosuyolu Heart and Research Hospital, Istanbul, Turkey; Kartal Kosuyolu Heart and Research Hospital, Istanbul, Turkey; Kartal Kosuyolu Heart and Research Hospital, Istanbul, Turkey; Kartal Kosuyolu Heart and Research Hospital, Istanbul, Turkey; Kartal Kosuyolu Heart and Research Hospital, Istanbul, Turkey; Kartal Kosuyolu Heart and Research Hospital, Istanbul, Turkey

**Keywords:** carotid endarterectomy, coronary artery disease, hypothermia

## Abstract

**Purpose:**

We sought to determine whether hypothermia provided any benefit in patients undergoing simultaneous coronary artery bypass graft surgery (CABG) and carotid endarterectomy (CEA) using one of two different surgical strategies.

**Methods:**

Group 1 patients (*n* = 34, 88.2% male, mean age 65.94 ± 6.67 years) underwent CEA under moderate hypothermia before cross clamping the aorta, whereas group 2 patients (*n* = 23, 69.6% male, mean age 65.78 ± 9.29 years) underwent CEA under normothermic conditions before initiating cardiopulmonary bypass (CPB). Primary outcome of interest was the occurrence of any new neurological event.

**Results:**

The two groups were similar in terms of baseline characteristics. Permanent impairment occurred in one patient (2.9%) in group 1. One patient from each group (2.9 and 4.3%) had transient neurological events and they recovered completely on the sixth and 11th postoperative days, respectively. Overall, there was no statistically significant difference between the two groups with regard to occurrence of early neurological outcomes (*n* = 2, 5.8% vs *n* = 1, 4.3%, *p* = 0.12).

**Conclusions:**

This study could not provide evidence regarding benefit of hypothermia in simultaneous operations for carotid and coronary artery disease because of the low occurrence rate of adverse outcomes. The single-stage operation is safe and completion of the CEA before CPB may be considered when short duration of CPB is required.

## Abstract

The co-existence of coronary, carotid, peripheral and renal atherosclerotic diseases is not infrequent and it was reported that 24% of patients with coronary artery disease have at least one additional atherosclerotic lesion.^1^ In previous studies, 4.6 to 8.0% of patients with coronary artery disease (CAD) had severe coronary artery stenosis (CAS), the extent of the atherosclerotic involvement being significantly correlated with the carotid and coronary arteries.^2^,^3^ Simultaneous surgical management of concomitant coronary and carotid artery disease has been the focus of interest in the past two decades since success rates of coronary artery bypass grafting (CABG) has substantially increased while a preventive approach for adverse neurological outcomes has gained popularity.^4^ Carotid stenosis and previous history of cerebrovascular disease were reported to be among the most prominent risk factors for peri-operative stroke and neurocognitive decline in patients undergoing CABG.^5^

The optimal decision for the timing of carotid endarterectomy (CEA) is controversial in patients submitted for CABG since data focusing on establishing the best strategy of practice are limited.^6^ There have been numerous cross-sectional studies reporting favourable outcomes for both simultaneous and staged CEA and CABG procedures,^7^-^9^ and some authors have suggested that the decision to perform the two procedures simultaneously should be made based on strict patient selection criteria.^10^ Nevertheless, delaying the CEA was found to be an independent predictor of early stroke and death in one recent randomised trial.^11^ This uncertainty led to an increasing trend towards individualisation of the treatment in patients with concomitant disease.

Some earlier studies implied the potential role of hypothermia as a preventative measure against adverse postoperative outcomes in patients undergoing single-stage on-pump CABG and CEA.^12^,^13^ However, these studies fell short of their goal of determining whether hypothermia provides protection, because none of them involved a control group of patients undergoing CEA under normothermic conditions. In this study we sought to determine whether hypothermia provided any benefit in patients undergoing simultaneous CABG and CEA using one of two different surgical strategies.

## Methods

This retrospective cohort study was undertaken in a single tertiary educational hospital and was made up of 57 patients who underwent concomitant CEA and CABG between 2006 and 2013. Patients’ archived records, counselling charts and laboratory tests were reviewed in January 2013.

Patients were divided into two groups. Group 1 patients (*n* = 34, 88.2% male, mean age 65.94 ± 6.67 years) were those undergoing CEA under moderate hypothermia, after initiation of cardiopulmonary bypass (CPB) and before cross clamping the aorta, whereas group 2 patients (*n* = 23, 69.6% male, mean age 65.78 ± 9.29 years) were those undergoing CEA under normothermic conditions before initiation of CPB.

According to our institution’s policy, patients scheduled for CABG undergo duplex ultrasound scans for screening of CAS, and those with ≥ 70% stenosis and/or plaque ulceration in at least one carotid artery undergo carotid angiography. It was the surgeon’s discretion which procedure would be performed in any given patient during the study period, but this decision was not made on characteristics or certain risk factors that the patient had.

Patients undergoing emergency operation, multiple interventions including valve, ascending aorta and left atrial size reduction were excluded. Patients with a history of recent cerebral infarction, transient or permanent ischaemic stroke and/or cerebral bleeding were also excluded.

Daily neurological assessment by physicians of patients undergoing CEA has been the standard of care in this institution. Daily chart notes were carefully inspected for records regarding neurological status of the patients. Based on these observations, the primary outcome of interest was considered occurrence of any new neurological event, including seizures, coma and/or ipsilateral or contralateral motor or sensorial involvement during the postoperative period. Beginning on the day after the operation, all patients were given subcutaneous low molecular weight heparin and aspirin until discharge home.

All operations were performed under general anaesthesia. In both groups, common, internal and external carotid arteries were exposed first through a standard approach anterior to the sternocleidomastoid muscle. In group 1, a median sternotomy was made, the internal thoracic artery was harvested, 350 UI/kg of systemic heparin was administered and cardiopulmonary bypass was established in a standardised fashion.

The carotid arteries were clamped when the systemic temperature had cooled down to moderate hypothermia. CEA was performed, clamps were removed and the neck incision was left open until heparin reversal by applying sponges over the wound. The surgeon then cross clamped the aorta and proceeded with the CABG procedure in a standardised fashion.

In group 2, systemic heparin was administered in similar doses and CEA was performed before the sternotomy was made. The clamps were removed and the neck incision was left open by applying sponges. The surgeon proceeded with a median sternotomy and the standardised CABG procedure thereafter.

In both groups, the arteriotomy was closed primarily in all patients without using intraluminal shunts. Cardiac arrest was established by antegrade normothermic blood cardioplegia and proximal anastomoses were performed with an aortic side-biting clamp.

## Statistical analysis

Statistical analyses were performed using SPSS 19.0 packaged software. Normal distribution of variables was tested using visual histograms and the Kolmogorov–Smirnov test. Descriptive statistics for continuous variables were reported as mean ± SD and descriptive statistics for categorical variables were reported as frequency and percentage. Categorical variables were compared using the chi-square or Fisher’s exact tests, where appropriate. Continuous variables were compared using the independent samples *t*-test. Since new neurological events occurred in very few patients, no additional tests (univariate or multivariate analysis) were performed to identify independent predictors of adverse outcomes. Study power was tested using G-power software.

## Results

Baseline patient characteristics were similar between the two groups (Table 1). The mean time of CPB was shorter and mean level of hypothermia was lower in group 1 patients than in those in group 2. There was no difference between the two groups regarding other operative values (Table 2). A total of 28 patients (82.3%) in group 1 and 18 patients (78.2%) in group 2 were asymptomatic for neurological complaints (*p* = 0.76).

**Table 1:** Baseline characteristics

*Variables*	*Group 1 n (%)*	*Group 2 n (%)*	*p*
Men	30 (88.2)	16 (69.6)	0.09
Age	65.94 ± 6.67	65.78 ± 9.29	0.9
Previous MI	9 (26.5)	6 (26.0)	0.97
Unstable angina	3 (8.8)	1 (4.3)	0.64
Previous CVA	11 (32.3)	5 (21.7)	0.48
Stroke	4 (11.8)	1 (4.3)	0.63
Hypertension	29 (85.3)	18 (78.3)	0.50
Diabetes	17 (50)	12 (52.2)	0.87
Hyperlipidaemia	19 (55.9)	9 (39.1)	0.21
Renal failure	4 (11.8)	1 (4.3)	0.63
Smoking	27 (79.4)	14 (60.9)	0.12
PAD	13 (38.2)	7 (30.4)	0.54

MI; myocardial infarction, CVA: cerebrovascular accident, PAD; peripheral arterial disease.

**Table 2:** Operative variables

*Variables*	*Group 1 n (%)*	*Group 2 n (%)*	*p*
Number of bypass grafts	2.9 ± 0.6	2.9 ± 0.8	0.91
CPB time (min)	72.3 ± 21.9	59.6 ± 20.8	0.03
Time of cross clamping (min)	32.6 ± 9.4	31.2 ± 6.9	0.78
Carotid clamping time (min)	9.8 ± 2.7	9.7 ± 3.1	0.91
Hypothermia	30.3 ± 1.3	35.8 ± 0.7	0.001
Left CEA, n (%)	17 (50)	14 (61)	0.41

CPB; cardiopulmonary bypass, CEA; carotid endarterectomy.

Overall, early mortality occurred in one patient from each group (*n* = 2, 3.7%, *p* = 0.86). Both patients died of low cardiac output syndrome. Adverse neurological outcome with permanent impairment occurred in one patient (2.9%) from group 1. One patient from each group (2.9 and 4.3%) had transient neurological events but they recovered completely on the sixth and 11th postoperative days, respectively.

Overall, there was no significant difference between the two groups with regard to occurrence of early neurological outcomes (*n* = 2, 5.8% vs *n* = 1, 4.3%, *p* = 0.12) (Table 3). None of the patients had revision for bleeding, cardiac tamponade, low cardiac output syndrome, arrhythmia and systemic, respiratory or wound infection. The length of intensive care unit stay and hospital stay were similar between the two groups (Table 3).

**Table 3:** Postoperative variables

*Variables*	*Group 1 n (%)*	*Group 2 n (%)*	*p*
Early neurological outcomes	2 (5.8)	1 (4.3)	0.12
Intensive care unit stay (day)	2.9 ± 1.7 (1–9)	3.1 ± 2.2 (1–11)	0.21
Hospital stay (day)	5.2 ± 4.5 (4–8)	5.3 ± 4.2 (4–9)	0.19

Because of the limited sample size (34 vs 23 patients) and very low occurrence rate of neurological events, the study power (1-β error) was quite low (< 25%) in this study to provide evidence for rejection of the null hypothesis. When significantly shorter mean CPB time in group 2 than in group 1 patients (59.6 ± 20.8 vs 72.3 ± 21.9) was considered as a secondary outcome, our study had 71% power (1-β error) with an α error of 0.05 and an estimated effect size of 0.60 (medium effect size).

## Discussion

Our study showed that single-stage CEA and CABG is safe and carotid intervention may be performed either before or after initiation of CPB without adding much more complexity to the procedure. However, because of the low effect size, which is a direct measure of the occurrence rate of the outcomes, our study could not draw a definitive conclusion regarding the protective effect of hypothermia during CEA.

The decision whether each patient would receive CEA under hypothermia or not was the surgeon’s discretion in general, and this decision was not dependent on objective criteria. However, it could not be totally neglected that the surgeon might preferentially have intended to take the short CPB time into consideration and performed CEA initially in the presence of certain conditions or features, such as patient fragility or poor coronary arterial structure. These features were not taken into consideration as potential risk factors since patient records were not standardised to provide objective information.

In addition, the majority of our patients were asymptomatic of neurological complaints, which was another known risk factor for the development of neurological events after simultaneous CEA and CABG surgery. The operations were performed within a period of seven years, in which the standard of surgical approach did not undergo modification by the surgeon.

Similar to ours, in one earlier study where Di Tommaso *et al.*12 performed 73 combined CEA and CABG procedures, the occurrence rate of temporary neurological deficits was quite low (five patients), and an additional six patients had cerebrovascular events during the late follow up. These authors attributed the low rate of neurological complications to CPB-related benefits, including haemodilution, pulsatile flow and hypothermia.

Yildirim *et al.*^14^ used a similar technique for single-stage operation and reported that four of 72 patients had neurological complications and two of these became permanently disabled. In a report on a small series of patients, neurological events occurred in one of 15 patients undergoing combined CEA and CABG under mild hypothermia.^15^ Finally, Sadeghi *et al.*^13^ reported no early neurological outcomes and only one stroke during follow up when CEA was performed under mild hypothermia.

Although all these studies have implied that hypothermia is likely to have cerebral protective effects, our results and those of others suggest that the lower complication rates may not only be produced by the effect of hypothermia but may also be related to the surgeons’ cumulative experience with the CEA technique.

Darling *et al.*^16^ reported in their series of 420 patients that the risk of stroke was 1.2% and mortality was 2.4% in patients undergoing CEA prior to CABG. Another study showed that rate of major stroke was 3.3%, whereas transient neurological deficit rate was 9.9%^17^ in 30 patients undergoing CEA before CABG. Santos *et al.*^7^ recommended a < 30-day peri-operative period between initial CEA and CABG. Although some authors^10^ have suggested that the indication for performing combined CEA and CABG should be restricted to patients requiring urgent CABG, we believe that a single-stage operation would often be the desired option both for the surgeon and the patient, provided that there would be no additional risks using a combined technique.

We are aware that our study had many limitations, including retrospective design, lack of randomisation, single institution experience and small sample size. Also, patients in this study were not divided into treatment arms based on objective criteria, a fact that may be a source of potential bias. Finally, followup information was not sufficient to report since the majority of patients could not respond to our invitation for follow up because they lived in distant regions of the country.

## Conclusions

Low rate of adverse neurological outcomes after simultaneous CABG and CEA under CPB were previously attributed to hypothermia by a possible mechanism of reducing neuronal ischaemia. However, our study and previous ones showed that it is difficult to establish such a relationship due to low occurrence of adverse outcomes after combined operations. Single-stage operation is effective and safe, and performing the CEA before initiation of CPB may be considered when short duration of CPB is required.
